# Ethics Considerations in Global Mobile Phone-Based Surveys of Noncommunicable Diseases: A Conceptual Exploration

**DOI:** 10.2196/jmir.7326

**Published:** 2017-05-05

**Authors:** Joseph Ali, Alain B Labrique, Kara Gionfriddo, George Pariyo, Dustin G Gibson, Bridget Pratt, Molly Deutsch-Feldman, Adnan A Hyder

**Affiliations:** ^1^ Berman Institute of Bioethics Johns Hopkins University Baltimore, MD United States; ^2^ Department of International Health Johns Hopkins Bloomberg School of Public Health Baltimore, MD United States; ^3^ Nossal Institute for Global Health School of Population and Global Health The University of Melbourne Melbourne Australia; ^4^ Gillings School of Global Public Health The University of North Carolina at Chapel Hill Chapel Hill, NC United States

**Keywords:** ethics, mobile phone survey, mHealth, noncommunicable diseases, research ethics, bioethics

## Abstract

Mobile phone coverage has grown, particularly within low- and middle-income countries (LMICs), presenting an opportunity to augment routine health surveillance programs. Several LMICs and global health partners are seeking opportunities to launch basic mobile phone–based surveys of noncommunicable diseases (NCDs). The increasing use of such technology in LMICs brings forth a cluster of ethical challenges; however, much of the existing literature regarding the ethics of mobile or digital health focuses on the use of technologies in high-income countries and does not consider directly the specific ethical issues associated with the conduct of mobile phone surveys (MPS) for NCD risk factor surveillance in LMICs. In this paper, we explore conceptually several of the central ethics issues in this domain, which mainly track the three phases of the MPS process: predata collection, during data collection, and postdata collection. These include identifying the nature of the activity; stakeholder engagement; appropriate design; anticipating and managing potential harms and benefits; consent; reaching intended respondents; data ownership, access and use; and ensuring LMIC sustainability. We call for future work to develop an ethics framework and guidance for the use of mobile phones for disease surveillance globally.

## Introduction

Routine public health surveillance, a continuous process of collecting and analyzing health-related data, is critical for monitoring disease epidemiology and implementing public health programs [1]. Traditionally, active surveillance has often been carried out through face-to-face household surveys or contact with health care providers to obtain relatively reliable information [1,2]. This manner of data collection can be considerably resource-intensive [2]. In recent years, interest in alternative and streamlined approaches has grown [3]. In particular, some have begun to explore the use of basic mobile phone surveys (MPS) to augment data collection, anticipating several potential advantages over traditional methods, and leveraging existing and emerging mobile survey platforms [3,4]. MPS may provide opportunity for data collection more quickly, with fewer staff and lower overall programmatic costs; though approaches are only now being optimized and compared with traditional surveillance methodologies [5-7].

There is particular interest in using MPS in low- and middle-income countries (LMICs) where household surveys can be challenging to implement and frequent data collection is critical to effective monitoring of rapidly changing health behaviors and disease burdens [5]. Interest in MPS technology utilization in LMICs has also grown in large part due to the expansion of mobile phone use within such countries over the past decade. In sub-Saharan Africa, mobile phone ownership among individuals aged over 15 years reached 69% in 2015 [8]. Such increases present a considerable opportunity to access previously hard-to-reach populations; however, research has also documented some persistent inequities in mobile phone ownership, access, and use along axes of gender and income level, which may complicate the collection of representative data and make it difficult to ensure the equitable distribution of potential harms and benefits from research or surveillance [9-11].

In most wealthy countries where smartphones and reliable high-speed data networks are prevalent, mobile phone apps are also increasingly being used for health promotion, management, and surveillance [12]. However, the use of these technologies and approaches is more limited in LMICs where smartphone ownership and use is relatively low; in 2015, 37% of adults in low-income countries reported owning a smartphone, compared with 68% of adults in wealthy countries [8]. Challenges such as poor access to the Internet, cost, as well as lack of familiarity with technology limit the utility of smartphone-based health assessments across diverse LMIC populations [13,14]. Thus, emerging health surveillance programs in many LMICs typically have sought to conduct surveys using approaches conducive to simple cellular phones and networks, such as short message service (SMS), interactive voice response (IVR), and computer-assisted telephone interviews (CATI).

A relatively small number of studies have been conducted to determine the effectiveness of mobile phone–based disease surveillance in LMICs. These studies have focused on both infectious and chronic diseases, the latter of which is of particular interest as rates of noncommunicable diseases (NCDs) are on the rise within LMICs [15,16]. Researchers conducted pilot studies on the penetration of mobile phones and the use of mobile health (mHealth) tools for NCD surveillance and care in several countries, including Bolivia and South Africa [13,16]. Although many advocate for increased use of mHealth for NCD monitoring and care, rigorous research is necessary to inform practice. A research framework, developed by Bloomfield and colleagues [16] for advancing mHealth technology, to help address NCDs specifically in sub-Saharan Africa can help guide future activities as interest expands.

Additional recent efforts are ongoing to identify and navigate the corresponding ethics-related issues that arise when planning and implementing MPS in LMICs. Previous work has detailed several of the central ethics issues facing mHealth in general [11]. Vayena and colleagues [17] have specifically begun to map the ethical issues in “digital disease detection” or “digital epidemiology,” particularly when “big data” present the opportunity to aggregate digital information from multiple existing sources to, for example, identify potential disease outbreaks. However, much of the focus of emerging digital and mHealth health ethics literature is on the challenges associated with complex data systems, and the use of smartphones, tablets, and other more advanced mobile technologies.

Efforts have certainly also been made to guide “traditional” public health surveillance programs in LMICs, including within the International Network for the Demographic Evaluation of Populations and Their Health (INDEPTH) Network, a collaboration of health and demographic surveillance systems across 49 field sites in 20 countries [18]. INDEPTH has published a Resource Kit in an effort to advance best practices for surveillance; however, the kit only briefly touches on ethics considerations [19]. Thus, despite the growing use of mobile phone–based surveys and surveillance programs in LMICs, including for NCDs, in-depth analyses of relevant ethics challenges are quite limited.

With support from the Bloomberg Philanthopies’ Data for Health Initiative, we initiated a project to critically examine the ethical, legal, and societal issues associated with the use of MPS for NCD risk factor surveillance in LMICs [20]. The project involves activities to optimize mobile phone surveys and platforms in order to expand the capabilities of NCD risk factor data collection [4], presenting a unique opportunity to explore, both conceptually and empirically, the ethics challenges first hand.

In this paper, we provide an initial conceptual review of several central ethics challenges that ought to be considered when formulating and administering MPS of NCD risk factors in LMICs. The issues discussed in this paper reflect both key questions that have emerged during our own initial programmatic efforts, as well as those that are likely to become relevant as MPS efforts expand. The ethics issues raised in this manuscript were reviewed by diverse group of approximately 30 global experts in ethics, mHealth, social science, health policy, MPS technology, and regulatory oversight during a technical workshop. Workshop participants provided valuable feedback that helped to refine our analyses and focus efforts on the central challenges. The ethics-related issues that we discuss are mainly presented based on when they occur in the MPS process—before data collection, during data collection, and after data collection—acknowledging that some of the issues raised (such as stakeholder engagement and risk-benefit assessment) are cross-cutting (see Table 1). We discuss relevant considerations for each ethics issue, explore broader societal issues, and make recommendations for future work.

**Table 1:** Suggested ethics consideration in mobile phone survey (MPS) of noncommunicable disease (NCD) risk factors by phase.

Issues		Key considerations or questions	Available options (indicative)
**Before data collection**			
	Defining the activity	Does the activity constitute research, M&E, and/or surveillance?	Nature of activity is defined by independent persons
		Who defines the nature of the activity?	Criteria to distinguish types of MPS^b^ are established and applied
		What ethical requirements follow? IRB^a^ or other review? Disclosures or authorizations? Transformation of data into policy, practice?	
	Stakeholder engagement	What are the ethical goals of engagement?	Identify local norms and practices associated with the use of mobile phones
		Who should be engaged?	Determine the social, cultural, legal, and public health significance of the information being collected
		How and when should stakeholders be engaged?	Identify stakeholders, for example, technical experts, community representatives, health system agents, donor agency representatives
	Appropriate design	How should the survey be delivered? What qualities of survey delivery will affect the respondent and data validity? What are the consequences of: IVR^c^ or CATI^d^ or SMS^e^ or mixed modality? Will the survey reach minority, disadvantaged, marginalized groups?	Align delivery method with population characteristics, for example, if low literacy, IVR may be preferred
		Does the delivery method influence social desirability bias?	Use all major languages commonly used in other traditional surveys
		Linguistic strategy: how many and which languages ought to be used to administer the survey?	
	Anticipating potential harms and benefits	What are the potential burdens or risks of participating in a MPS program (at the individual, community, national levels)? For example, Sensitive information and stigma	User-testing, interviews, and focus groups to identify key sensitive issues and design survey taking these into account
		What are the potential benefits or advantages of participating in a MPS program (at the individual, community, national levels)? For example, More frequent, less costly information, and better NCD^f^ care
		What strategies will minimize harms and maximize benefits?
		How will harms or benefits be balanced for disadvantaged and marginalized groups?
**During data collection**			
	Consent	What information about the MPS should be *disclosed* to respondents?	Disclose information consistent with *basic* consent elements
		How should respondents *authorize* their participation?	Review local practices, population preferences, and legal permissibility of different opt-in or opt-out approaches
		Should types of disclosure and authorization differ based on the MPS delivery method (IVR or CATI or SMS or mixed modality) and population?	
	Reaching intended respondents	Which MPS sampling and recruitment methods best reduce unnecessary burdens upon intended and unintended MPS recipients?	Take stock of other existing survey approaches in designing MPS delivery strategies
		What approach to MPS incentive delivery is most appropriate?	Conduct follow up surveys by human caller on limited sample to verify
		Are existing laws likely to make it difficult to reach respondents using MPS?	Consult local regulatory authorities to identify and address potential areas of concern
**After data collection**			
	Data ownership, access and use	To whom do the data belong?	Establish advance agreements to guide terms of data ownership, access, and use
		What data can be shared?	Consider restrictions on data access and use for purposes that do not advance public health
		Who should have access to the data?	
		How should the data be shared?	
		To what uses can the data be put?	
	Ensuring LMIC^g^ sustainability	What are different actors’ responsibilities to promote and advance local sustainability?	Agree upon sustainability and capacity development commitments in advance
		Who is responsible for deciding who is allocated which strategies to undertake?	Identify core areas of local need and strength
		Should ongoing MPS efforts be supported only in the original data collection area or more broadly?	Integrate with existing programs and approaches where available

^a^IRB: institutional review board.

^b^MPS: mobile phone survey.

^c^IVR: interactive voice response.

^d^CATI: computer-assisted telephone interview.

^e^SMS: short message service.

^f^NCD: noncommunicable disease.

^g^LMIC: low- and middle-income country.

## Before Data Collection

### Defining the Activity

MPS are being rolled out across LMICs to determine their potential for collecting NCD risk factor information, with a long-term goal of generating reasonably reliable and valid population representative data. Therefore, in addition to collecting individuals’ responses to questions about NCD risk factors such as diet, physical activity, tobacco or alcohol use and the like, the surveillance approach is itself being actively monitored, studied, and improved. Understanding the circumstances under which MPS activities constitute research, program monitoring and evaluation (M&E) and/or public health surveillance can be important to determining the nature of ethics and regulatory oversight typically required.

It can be difficult, however, to characterize the nature of particular MPS public health data collection activities, especially where multiple overlapping purposes exist and activities are implemented with significant involvement of many different types of stakeholders, including public health agencies and policy makers. Moreover, in contrast with research, ethical practice norms for M&E and surveillance are less established in general, let alone in the context of MPS. Therefore, even once MPS approaches for monitoring NCDs and other risk factors have been reasonably optimized through research and data can be considered valid, questions about ethics, and oversight requirements for ongoing public health use of MPS will likely remain. It is furthermore imperative to understand how the intent of any MPS, regardless of how surveyors define it, is perceived by respondents. Many mobile phone users may associate phone surveys with marketing and commercial data collection.

We suggest that defining the nature of the activity encompasses three central questions: (1) what features are relevant to determining whether a MPS activity constitutes research, M&E, and/or surveillance; (2) who defines the nature of the activity; and (3) what ethical requirements follow for each type of MPS activity [21]? Although it is beyond the scope of this paper to answer these questions fully, some believe that the proper identification of the nature of the activity is highly relevant to, among other things, determining whether the activity undergoes institutional review board (IRB) or other forms of prospective ethics review, the nature of disclosures and authorizations provided to and obtained from respondents, and the extent of the obligation to transform data into policy and practice [22]. On the other hand, others may argue that the tendency to link key ethics and regulatory oversight requirements to “labels” (such as research, M&E, and surveillance) can at times distract from the need to consider the nature of potential harms and benefits, and their distribution across society, irrespective of what the activity is called. It seems appropriate, particularly at early stages of MPS development and utilization for NCDs, that care be taken not simply to rely on interested parties to define the nature of applicable ethics and regulatory requirements. Independent consult from ethics and regulatory experts should be sought, especially from within countries where MPS will be implemented.

### Engagement With Local Stakeholders

Although MPS of NCD risk factors can be rolled out relatively easily and from a single or cyber location to the remotest regions of the globe, this does not in any way decrease the need for local engagement and indeed partnership. In fact, robust engagement seems imperative for the effective use of MPS for NCD risk factor surveillance in LMICs given the significant potential for over-utilization of MPS capabilities, sensitivity (legal, political, financial, and social) to perceived mobile phone “spam,” and broader goals relating to local recognition and uptake of data. Three key questions related to stakeholder engagement in NCD MPS programs require attention: (1) what are the ethical goals of engagement in a MPS program; (2) who should be engaged and why; and (3) how and when should stakeholders be engaged?

On the *first* question, the *ethical goals* of MPS engagement may be conceived to include (1) understanding local norms and practices associated with the use of mobile phones and aligning approaches as best as possible with local expectations to minimally disturb respondents and other stakeholders such as mobile network operators; and (2) identifying the social, cultural, legal, and public health significance of the NCD risk factor information being collected to anticipate and mitigate avoidable informational risks and maximize potential benefits. Pursuit of these goals can help ensure local desirability, relevance, representativeness, and sustainability of the program. They imply a continuous engagement process inclusive of collaborative planning, implementation, and capacity strengthening.

It quickly becomes evident that attention to the *goals* of engagement in the context of a particular MPS effort is also critical to the process of identifying *who* to engage and *how* to do so. For example, ensuring *relevance* and *representativeness* of an NCD MPS effort would encompass the goals of deciding collaboratively on which diseases, and risk factors and from whom to gather data. The former would entail assessing which NCDs are of high burden or priority in the particular country or locale being surveyed and ensuring that the diseases surveyed included those of burden to disadvantaged and marginalized groups within the area. The latter would entail deciding what subpopulations to rely on to collect data and ensuring that they include better-off *and* disadvantaged areas or groups. If the population from which data are collected is not broadly representative or does not include sufficient numbers of different marginalized groups, the capability to identify the *distribution* of the burden of disease will be restricted. Key questions associated with access and demographics, such as who has the hardest time owning mobile phones, who has the hardest time accessing mobile phones, and what norms promote inequity of access to mobile phones, are crucial to answer in advance, when possible [11]. Those who are likely to be underrepresented by MPS can potentially be included through other more suitable means of data collection identified through direct engagement with those populations or their representatives. Both informal and formal engagement processes can serve these purposes.

Similarly, local *desirability* and *sustainability* considerations might require that individuals pursuing new MPS programs in low-resource settings also consider the local interest in and capacity to carry out the program. Are existing technologies or programs within the country being appropriately leveraged? What types of data for specific groups may be captured through MPS that have not been captured with household surveys or other surveillance methods? Who should coordinate and implement the program locally, alongside external or government actors? Is the program being externally imposed, using an infrastructure that cannot be supported with local resources?

The comparative ease with which technology and mobile devices can now be used to collect and transmit data has also sparked debate globally regarding the proper use of such technology and information for public and private purposes. Negative public sentiments toward electronic surveillance in other arenas (eg, for national security, intelligence or commercial purposes) may transfer to disease surveillance and require concerted efforts to overcome. This is a critical societal issue which requires broader discussions around the perceived and actual risks of collecting and using data acquired through information technology to improve the public’s health. Establishing awareness campaigns and fora for open discussion may increase understanding and trust in MPS, particularly in locales where the approach is new or where it has previously been “abused.” Addressing any such perceptions and questions is important for ensuring that the MPS program is accepted and operates for the benefit of all stakeholders.

### Appropriate Design

It is important to consider the various survey delivery approaches (eg, SMS, IVR, and CATI) that can be used for MPS, and the possible consequences of each, particularly in terms of who they are likely to reach and whether this includes minority, disadvantaged, and marginalized groups within a particular country. As an ethics matter, any MPS ought to be conducted as efficiently and equitably as possible and in a manner that yields unbiased, reliable data. Therefore, one should prospectively consider which format of survey delivery is likely to provide such data in differing LMIC contexts.

SMS and IVR are likely to be lower cost and may reduce social desirability bias that can arise from face-to-face or phone interviews in which participants interact with other persons conducting the surveys [23]. IVR strategies may be more flexible and increase respondent comprehension compared with SMS, particularly in LMIC settings where literacy levels may be an issue [3]. However, both SMS and IVR risk selection bias (groups, such as the elderly, may be poorly represented due to lack of familiarity with the interfaces), and misclassification bias if questions are not explained fully or responses are not entered properly. CATI offers the opportunity for a structured questionnaire to be delivered more personably, that is, by a live person who can offer basic clarifications over the phone; however, it is generally more time- and resource-intensive and can suffer from interviewer bias and the previously mentioned social desirability bias. Additionally, both CATI and IVR may introduce complex and potentially biasing elements between interviewer and respondent due to cultural or demographic differences as interpreted through voice and lexicon. Weighing the pros and cons of different types of survey delivery methods, including their accessibility to disadvantaged and marginalized groups, is important to the ethical goal of deploying MPS for the benefit of all, or at least not at the *systematic* disadvantage of some. Robust empirical comparisons of these modalities in the LMIC NCD context are therefore essential not only to the goals of surveillance, but also to comprehensive ethical analysis.

Similarly, all MPS programs, regardless of mode of delivery, must consider the proper languages and terminology to use. Selecting the best linguistic strategy is ethically important not only for obtaining representative data, but also for ensuring that participants understand all parts of the survey (including the reason why they are being surveyed). Many countries, particularly LMICs, have multiple languages and dialects; indeed, some countries like Indonesia and Nigeria have hundreds [24,25]. Often times “official” languages are most representative of dense urban areas where health burdens and behaviors differ from more rural areas. Variations between written and spoken language are also very common and the process of developing and verifying survey text and audio recordings in many different languages is potentially time- and resource-intensive. Although similar challenges may apply to traditional face-to-face household surveys of NCDs, the use of visual aids (show cards) and local data collectors with regional language skills can support data collection across varying linguistic areas. Where MPS are distributed using random digit dialing (RDD)—an approach that is of particular interest for nationally representative surveys given relative ease of implementation and statistical advantages—building in adequate linguistic representation into MPS is key.

### Anticipating Potential Harms and Benefits

Anticipating potential harms or burdens and benefits or advantages is a critical part of MPS planning. This may involve different types of assessments, and the development of strategies to mitigate risk and maximize potential benefit. Those involved in collecting and storing data should always give due consideration to the risks (to individuals, communities, institutions, and nations) associated with the MPS approach and information being collected. Although the burden of participation may be relatively minimal, informational risks should be of concern to MPS for NCDs. For example, NCD risk factor data about alcohol consumption may be fairly sensitive in a country or region where consumption is culturally or religiously prohibited. Although certainly unacceptable to publicize individual-level data, even data showing particular communities to be associated with greater rates of alcohol consumption can also potentially generate harm.

Both those who have access to raw data and those who report findings have important data privacy and security-related responsibilities. MPS data are likely to be linked (at least in raw form) to individual phone numbers, even where a RDD approach is used. Whereas public telephone databases may not be available in many LMICs, mobile network operators and government authorities who may be engaged in MPS usually have access to sufficient information to potentially identify many individuals, though are unlikely to have reason to do so. Indeed, in some instances, for example, with data collected via SMS, both incoming and outgoing data itself may be automatically recorded by mobile network operators alongside personally identifying information. Planning is needed to develop protocols that define legitimate data use and protect against informational risk. Technical MPS platform developers or intermediaries should also be engaged to support alignment of software capabilities and data management practices with risk mitigation strategies.

Potential harms, however, should not be considered in the abstract and merely prospectively. They must be assessed in relation to potential *benefits*, and the *actual* accrual of harms and benefits must be monitored during MPS and evaluated afterwards. Potential population-level *benefits* of a well-designed and implemented MPS of NCD risk factors may include the opportunity for more frequent, less resource-intensive, and more convenient data collection across large geographic areas (not merely surveillance sites) to rapidly inform NCD policy and care. Another potential benefit of MPS, particularly IVR and CATI, may be its comparative advantage over face-to-face surveys in terms of respondent privacy. Other individuals associated with or in proximity of MPS respondents are unlikely to know the details or context of the survey unless respondents choose to make this known. Still, SMS surveys may suffer from privacy breaches due to the “written format” of the survey, unlike the digital and voice formats of IVR and CATI. Finally, MPS may yield better access to data from population subgroups that are harder to reach with household or other face-to-face survey methods.

Anticipating harms and benefits and evaluating the net risk of MPS therefore generally entails (1) identifying potential harms and benefits, and to whom they might accrue; (2) developing strategies prospectively to minimize harms and maximize benefits; and (3) assessing the balance of harms and benefits, again, including the balance for disadvantaged and marginalized groups. Although we do not describe processes for continuous monitoring and evaluation of harms and benefits in this manuscript, we emphasize its overall importance for MPS. Importantly, we neither believe that there is sufficient evidence to justify a *presumption* of net harm or benefit of MPS, nor do we think it wise to make such a generalization. Rather, the potential harms and benefits of each MPS program should be evaluated, ideally by independent personnel (eg, by an IRB) including individuals who represent those being surveyed. As part of this evaluation, where implementation strategies are unproven, the justification for testing the program in LMICs must also be assessed.

## During Data Collection

### Consent

The question of how to properly explain and obtain agreement to collect data using mobile technology has been raised in previous literature and is a potential challenge for MPS conducted in low-resource settings [11,26]. Here, we refer to a combination of basic *disclosure* and voluntary *agreement* as “consent” and distinguish it from a theoretical notion of “informed consent” which typically requires in-depth explanation of the data collection activity and understanding on the part of respondents before voluntary agreement. We suggest that obtaining *basic consent* of respondents is likely to be acceptable (and most practicable) for most forms of NCD risk factor MPS.

Approval of key “gatekeepers,” however, may also be important at a group level. For example, if not already centrally involved in initiating the MPS program, representatives from relevant governmental ministries and agencies (eg, Ministries of Health, Science and Technology, Telecommunications, Information Technology) should be fully briefed on the nature of the proposed MPS program and permitted to decide whether it aligns with relevant priorities, is allowed as a legal matter, would be an appropriate use of information communication systems and therefore whether it should proceed within the country. In instances where an MPS asks questions that are, for example, potentially stigmatizing for particular communities, or where data are only being collected from a more limited geographical zone, including local community representatives in decision-making is also likely important.

Although consensus has yet to form in defining the ethically required elements of consent *disclosure* for different types of MPS (eg, IVR, CATI, and SMS), a brief disclosure to respondents of essential MPS information might generally include the purpose, procedures, sponsor, key potential burdens, and benefits including expected duration and whether compensation (eg, airtime credits) will be provided, and the voluntary nature of the MPS. For multilingual MPS, it is important that this information be provided after respondents select a language of choice.

When indicating their *agreement* to participate, potential respondents can be requested to actively or passively o *pt-in,* or actively or passively *opt-out*. Examples of each are provided in Table 2. Of course, under all scenarios, the option to opt-out by not answering or hanging up the phone is available, at least for IVR and CATI. It remains unclear which approaches to authorization are *ethically* preferable for MPS of NCD risk factors and context may matter. If the various approaches to “demonstrating” agreement are determined to be roughly ethically equivalent in a particular context, then surveyors should follow the approach that is likely to yield the best response rate and thereby maximize the chance that data collected will be generalizable to the relevant population or subpopulation. This too has critical ethical importance as respondents are burdened unnecessarily when mHealth data sets are of little use.

### Reaching Intended Respondents

NCD risk factor data can be collected using MPS in a matter of hours or days, and as frequently as necessary, at least in principle. If conducted too frequently from the same respondents, public distaste for MPS may increase to a point where adequate completion rates are unachievable. Regardless of frequency, it may be difficult to ensure that respondents actually meet inclusion criteria. This is of particular concern for RDD where anyone with a mobile phone number of a particular prefix can potentially receive a survey. For example, it is conceivable that an adolescent might respond to a MPS of adult risk factors.

It is ethically important to survey administration not to burden individuals unnecessarily. With MPS, one must account for individuals who have moved out of an intended survey locale, but are unintentionally sampled because they have a phone number prefix for the intended area. Furthermore, LMIC mobile networks often have quality and cost issues leading many individuals to obtain subscriber identity module (SIM) cards or phone numbers for multiple networks. This makes it possible that some may receive and perhaps respond to a survey multiple times. Many of these challenges could also cause problems primarily for data reliability and validity, but with careful design, sampling, and statistical analyses, most of these challenges can be managed, as a technical matter [27].

In order to encourage survey completion and accommodate any financial burdens, it is increasingly customary to provide respondents with phone airtime credits. Although many have debated whether and when such incentives can be excessive or inadequate, considerations relating to the timing of incentive delivery may also raise ethical and data quality considerations. Incentive delivery upon MPS completion may increase the likelihood that individuals complete the entire survey; however, some may provide false data, for example, indicate a false age or press random digits, to reach completion. This approach also does not accommodate those who, for reasons not under their control, were only able to partially complete a survey. Providing some airtime credits to all respondents regardless of survey completion status may mitigate these risks and be “fairer,” but will likely result in greater overall expense and perhaps decrease the chance that respondents will answer all questions. It remains to be determined empirically whether these particular concerns are, in general, of greater significance to MPS as compared with many face-to-face surveys. Certainly any known measures to reduce these complications should be used.

It may be more difficult to “design around” other issues which have ethics or regulatory implications. For example, it may be challenging to roll out a MPS using RDD in a country where telecommunication law prohibits “robo-calling” except when done by certain agents for the purpose of public service and safety messaging. Other countries may prohibit “masked” or “restricted” phone calls where a call is made through an intermediary and the caller remains hidden. Data collectors may need to work with government authorities in such cases to identify workarounds that still honor the principles embodied in the restriction, for example, establish a call-back number that provides an automated “hotline” with additional information about the MPS.

**Table 2:** Mobile phone survey (MPS) consent authorization options and examples.

Options	Opt-in	Opt-out
Active	“press 1 if you would like to continue the survey”	“press 2 if you do *not* want to complete the survey”
Passive	“by completing this survey you agree to participate”	“the survey will automatically end if you do not respond to a question within 1 min”

## After Data Collection

### Data Ownership, Access, and Use

Perhaps the most important postdata collection question relating to the conduct of MPS for NCD risk factor surveillance is, “What will happen to NCD risk factor data once collected?” This can be reduced to at least five component questions: (1) to whom do the data *belong*, (2) *what* data can be shared, (3) who should have *access* to the data, (4) *how* should data be shared, and (5) to what *uses* can the data be put? Most of these questions can be deliberated, agreed upon, and clearly documented in advance in memoranda of understanding or contracts. However, where MPS programs involve multinational donors, LMIC and high-income country teams, and government agencies, such negotiations are likely to have ethical undercurrents relating to power imbalances and the abilities of particular partners to meaningfully engage in contract negotiation. Efforts to guide fair contracting, such as the Council on Health Research and Development (COHRED) Fair Research Contracting initiative may serve as useful references for MPS partnerships [28]. Data sharing agreements and models available from other public health surveillance systems can perhaps also serve as a guide for MPS agreements [29,30]. However, MPS may also give rise to additional opportunities and challenges for data sharing and use.

With MPS, large amounts of electronic data can, in theory, be aggregated, cleaned, analyzed, and shared relatively quickly. Opportunities to validate MPS NCD data against equivalent data should also be sought out. The degree of confidence in the quality and significance of the data will likely serve as an initial filter on what is shared and with whom. When data are found to be of sufficient quality, an obligation likely exists to feed the data back into the local health system so that there is an opportunity for it to inform priority setting and resource allocation. This raises questions such as, Should data be disseminated to all levels of the health system or just national authorities? Should they be disseminated to public and private actors in the health system? Should they be disseminated to those who responded to the MPS or the public more broadly? Do the World Health Organization or other global actors or donors have claims to information access and use?

Although perhaps primarily meant to inform the development of local interventions and policies to reduce NCDs, given the large amount of behavioral data that are likely to be generated through MPS of NCD risk factors, it is expected that various additional groups will be interested in obtaining and learning from the data. Nongovernmental organizations, insurers, large employers, urban planners, biotechnology companies, food manufacturers and distributors, and even alcohol and tobacco companies, to name a few, may all be eager to learn from NCD data and put them to uses that may or may not benefit the public’s health [30]. Should data stewards not plan and act in ways that demonstrate careful management of information collected, respondents or groups in LMICs ultimately may demand the ability to opt-in or opt-out of *particular* uses in the future—what is known as “specific consent”—or may refuse to provide their health information altogether [31]. Building and maintaining public trust in MPS is therefore highly critical to realizing the long-term potential of the approach globally. A review of experiences, challenges, and emerging best practices related to genetics and genomics research, biobanking and data sharing in LMICs may provide useful transferable lessons.

Finally, several additional questions relating to the use of data to support intervention and policy decisions are worthy of consideration. How should MPS findings factor into health priority-setting and resource allocation decisions made by policy makers in the context of other data and relevant considerations? Do host country actors (eg, ministries of health, district health officials) have an obligation to use the information generated from MPS surveillance to set or revise priorities and resource allocations within national and district health systems? For example, if the MPS identifies certain high NCD burdens for disadvantaged groups that are very poorly resourced within the district health system relative to other diseases, do district health officials have an obligation to shift resources to those diseases? Even if policy makers have an obligation to use MPS data to help set priorities, burden of disease is not the only (ethical) consideration in health priority-setting and resource allocation; there will be opportunity costs and other considerations involved in shifting resources. The question of how MPS data on NCD risk factors can and should be used by policy makers (and others) is, thus, quite complex and will entail consideration of many contextual features [32]. Where relevant, MPS data should at least feed into existing data-to-policy mechanisms that a given country may have.

### Ensuring Low- and Middle-Income Country (LMIC) Sustainability

We underscore the importance of local sustainability of MPS programs. Mobile phone penetration may be rapidly increasing in many LMICs, but the resources and technical capacity needed to independently develop, conduct, and analyze MPS are lagging behind due in part to the many challenges identified here and elsewhere [3]. To be sure, several countries have initiated their own health-related MPS programs with varying degrees of success and longevity. Those engaged in these programs are urged to continue to share case studies describing what is working, what is not, and what is needed to advance sustainability. Assessments of the ethical, legal, and societal dimensions of these programs are welcomed to better understand all contextual factors.

Implementing an unsustainable program is ethically questionable, as is the failure to consider sustainability aspects [33,34]. Determining the concrete contributions that all involved parties can make to promote and support sustained implementation is an important part of ensuring the ethical conduct of MPS. Again, this ethical dimension raises numerous unanswered questions: What are different actors’ (funders, survey implementers, local partners, ministries of health, etc) responsibilities to promote and advance sustainability? Do survey implementers have an obligation to initiate discussions with key stakeholders (at national, district, and local levels) about how the program can be sustained and to identify sustainability strategies (eg, advocacy, fund raising, capacity strengthening) in collaboration with those stakeholders? Do different actors then have a responsibility to carry out those strategies and, if so, on what basis? Who should be responsible for deciding who is allocated which strategies to undertake? Ought the program to be sustained in the areas where the initial surveillance implementation is being done or more broadly? These considerations, while easy to overlook, are vital to the success of public health information systems in LMICs.

## Recommendations and Conclusions

We have discussed many considerations in this paper which are in need of further conceptual and empirical exploration. These include identifying the nature of the activity; stakeholder engagement; appropriate design; anticipating and managing potential harms and benefits; consent; reaching intended respondents; data ownership; access and use; and ensuring LMIC sustainability. Identification of the degree to which existing ethics guidance in other arenas (eg, research ethics, generally) might support the navigation of ethics challenges associated with MPS for NCD risk factor surveillance in LMICs may be helpful. However, given the current lack of comprehensive ethics guidance for public health surveillance and for MPS in LMICs, several of the issues outlined above are likely to require fresh consideration.

Although we focus mainly on ethics issues in this paper, there is a need for a broad conceptual framework for the ethical, legal, and societal issues associated with MPS for NCD risk factors. Such a framework ought to include thorough analysis of various types of MPS activities (eg, research, M&E, and surveillance) and delivery methods (eg, IVR, CATI, and SMS). It would also likely benefit from empirical testing through application to ongoing MPS in LMICs. Empirical efforts to capture a cross-section of stakeholder perceptions relating to the identified challenges and any additional means to address them in practice would be of additional value.

Practical guidance relevant to the various stakeholders involved in designing, implementing, reviewing, funding, and overseeing MPS could then be formulated and updated as norms continue to develop and technological capabilities advance. It would be particularly useful for guidance documents to identify key issues, outline pros and cons of options available to stakeholders for each issue, review additional points to consider, and, provide references to resources relevant to each issue. In order to begin to address these needs, we hope to establish a global working group inclusive of experts in ethics, mHealth survey implementation, regulatory oversight and policy, public health, social science, and MPS platform development. We welcome opportunities to move forward in addressing these emerging issues collaboratively.

## Abbreviations

CATI: computer-assisted telephone interviews
COHRED: Council on Health Research and Development
INDEPTH: International Network for the Demographic Evaluation of Populations and Their Health
IRB: institutional review board
IVR: interactive voice response
LMICs: low- and middle-income countries
mHealth: mobile health
MPS: mobile phone survey
NCDs: noncommunicable diseases
RDD: random digit dialing
SIM: subscriber identity module
SMS: short message service
